# Machine learning for quantitative LIBS analysis of aluminum alloys: a comparison of random forest, gradient boosting, and extremely randomized trees

**DOI:** 10.1038/s41598-026-46449-2

**Published:** 2026-05-12

**Authors:** Aline Gonçalves Capella, Marta Martín López, Juan José de Damborenea González, María Ángeles Arenas, Ignacio Garcia Diego

**Affiliations:** 1 Institute of Science and Technology, Federal University of Sao Paulo, ICT-UNIFESP, Sao José dos Campos, 12242460 Brazil; 2 Department of Surface Engineering Corrosion and Durability, National Center for Metallurgical Research, CENIM-CSIC, Madrid, 28040 Spain

**Keywords:** Laser-induced breakdown spectroscopy, Machine learning, Regression models, Aluminum, Aluminum alloys, Engineering, Materials science

## Abstract

Laser-induced breakdown spectroscopy (LIBS) is a versatile technique for characterizing materials and analyzing elements, but it is limited in its quantitative accuracy due to nonlinear effects. This study uses machine learning (ML) regression algorithms to improve the quantification of commercially pure aluminum and its alloys with multiple alloying elements. A dataset of LIBS spectra from standard samples was used to train three tree-based ML models: Random Forest (RF), Gradient Boosting (GB), and Extremely Randomized Trees (ET). These models were evaluated using standard regression metrics. Prediction errors were consistently low for both in-sample (non-independent) validation and independent validation samples (MAE < 0.25 wt%), confirming strong generalization. The ET model produced the lowest errors, particularly for binary and ternary alloys. The GB model generated the most statistically indistinguishable results compared to the reference values, primarily due to higher prediction variance. The RF model showed intermediate performance. Reliable predictions required at least three representative samples per alloy family, which emphasizes the importance of dataset diversity in LIBS-based machine learning workflows. Therefore, integrating LIBS with ML is an effective strategy for predicting elemental compositions in metallic alloys. Future work will extend this approach to other aluminum alloys and support the development of scalable frameworks for quantitative analysis.

**Supplementary Information:**

The online version contains supplementary material available at 10.1038/s41598-026-46449-2.

## Introduction

Laser-induced breakdown spectroscopy (LIBS) is a technique that analyzes the elemental composition of a material by measuring the emission of excited atoms. In this method, a low-energy pulsed laser with high intensity (on the order of GW/cm²) vaporizes a small portion of the sample surface, creating a micro-plasma through atomization and ionization. The plasma then emits radiation, which a spectrometer collects and disperses, producing emission lines characteristic of the chemical elements present in the sample^1,2^. Hahn and Omenetto^3,4^ provided foundational insights into the plasma diagnostics and methodological strategies that define LIBS’s analytical capabilities. The authors demonstrated that LIBS offers several advantages, such as near-instantaneous results, minimal sample preparation, versatility, and operational simplicity. However, accurately quantifying samples with LIBS remains challenging due to several factors. Plasma parameters, such as electron density and temperature, influence line intensities and broadening. These parameters are also sensitive to experimental conditions and sample properties. Traditional methods, such as calibration-free laser-induced breakdown spectroscopy (CF-LIBS), derive concentrations directly from plasma emission spectra under the assumptions of local thermodynamic equilibrium (LTE), stoichiometric ablation, and optically thin plasma conditions. This approach requires careful plasma diagnostics, including determining the electron temperature (T_e_) via Boltzmann plots of emission line intensities and the electron density (n_e_) through Stark broadening analysis. Once these parameters are obtained, the relative elemental concentrations are computed by normalizing the transition probabilities and population densities derived from the measured spectra. Despite the advantages of CF-LIBS, such as rapid and minimally invasive analysis, the authors highlighted that achieving reliable compositional quantification continues to require careful plasma diagnostics and experimental design^3,4^. Furthermore, matrix effects, self-absorption, and surface oxidation, which are particularly prevalent in metallic systems such as aluminum alloys, can distort spectral features and reduce reproducibility^5^.

In this sense, machine learning (ML) algorithms are a prominent interdisciplinary tool for autonomously identifying relevant patterns in data. ML involves using algorithms and other methods on data sets to enable “learning” and predicting outcomes and classifying information. It is a subfield of artificial intelligence (AI)^6^. ML can be applied to LIBS data with little subjective intervention^7,8^. The method is based on LIBS calibration, which uses empirical correlations between emission line intensities and standards of known composition. This enables the calibration data to relate signal to concentration and learn multivariate and potentially nonlinear relationships across the entire spectrum in order to predict elemental composition.

According to Hao et al.^9^, machine learning (ML) can be divided into three categories: unsupervised, semi-supervised, and supervised learning. These categories are based on whether the data is labeled or unlabeled, and each category serves different analytical objectives. Unsupervised methods operate on unlabeled data to uncover intrinsic patterns without human intervention or predefined outputs. This makes them useful for exploratory analysis or when labeled data is scarce. In LIBS, for example, unsupervised methods have been used to cluster spectra from similar samples based on spectral similarity (e.g., K-means^10^ clustering and hierarchical clustering^11^ and to reduce the dimensionality of spectral data to improve visualization and facilitate further analysis (e.g., principal component analysis (PCA^2^ and ISODATA clustering^12^. Semi-supervised learning considers both labeled and unlabeled data by iteratively expanding pseudo-labels and making predictions on new data. It is important to note that semi-supervised learning uses labeled data to guide the learning procedure; thus, the method is only accurate when effective unlabeled samples are considered^13,14^. Supervised learning enables the mapping of features with predictive power through extensive calibration efforts using fully labeled datasets. Supervised patterns are used to train a model that can predict unknown samples. Several methods have been cited in the literature for predicting the concentration of elements in LIBS datasets based on the entire emission spectrum. These methods include multiple linear regression (MLR), partial least squares (PLS), the K-nearest neighbor method (KNN), support vector machines (SVM), artificial neural networks (ANN), and random forest (RF)^15–19^ and gradient boosting (GB)^20^.

RF regression has shown promise in processing LIBS datasets due to its ability to handle classification and regression tasks effectively and accurately while minimizing overfitting issues and processing quickly. LIBS datasets combined with the RF algorithm have been used for training sets and have demonstrated a relationship between the number of trees in the RF and the accuracy of sample classification^21^. Feng et al.^22^ showed that this combination can predict the degree of copper (Cu) pollution in atmospheric sedimentation samples without complicated sample preparation, providing a basis for pollution prevention and control measures. Liu et al.^23^ evaluated the quantitative analysis of toxic elements in polypropylene (PP) via LIBS coupled with RF regression. Their results showed that different preprocessing methods (normalization and mean centering) and the use of variable importance improved the method’s performance for plastic analysis.

Neiva et al.^20^ evaluated the integration of the SHapley Additive exPlanations (SHAP) algorithm with GB model to predict carbon (C) concentration in soils analyzed by LIBS, demonstrating a framework for decoding the complex interplay between emission lines and target elemental concentrations. Hu et al.^24^ performed a comparison between Light Gradient Boosting Machine (LGBM) and Standard Deviation (SD) methods to spectral data obtained via LIBS, showing a fast detection and quantification of the copper (Cu) and zinc (Zn) elements in aerosols. Compared to RF and GB, this algorithm introduces greater randomization in feature and split selection, making it more suitable for large scale datasets^25^. Ghazwani and Begum^26^ investigated the effectiveness of RF, GB, and ET (extremely randomized trees) models to predicting the solubility of the hyoscine drug and the density of solvents in the supercritical processing of pharmaceuticals, reporting R^2^ values above 0.96 with relatively low average percentage errors. Despite this, the application of the ET algorithm for LIBS prediction remains limited. A recent study by Guedes et al.^27^ proposed a LIBS-based for carbon (C) quantification in soil, employing SHAP to guide variable selection and using ET model for training on a refined dataset. The results demonstrated superior accuracy and robustness across training and validation sets, highlighting its effectiveness in handling spectral complexity and its lower susceptibility to data variability. Table 1 presents a comparison of tree-based models for elemental quantification using LIBS.

LIBS presents specific challenges when used on aluminum alloys which can affect the stability and the accuracy of ML-based quantification. These challenges include the high thermal conductivity of aluminum, low ablation limits, and spectroscopic phenomena. These phenomena include self-absorption in the aluminum (Al), resonant lines, and overlap with lines from alloying elements (e.g., Cu, Zn, and Mg)^28^. S. Van den Eynde et al.^29^ explored the use of quantitative LIBS for characterizing aluminum alloys in recycling and manufacturing. The authors showed that accurately determining minor alloying elements in aluminum alloys is difficult due to matrix effects, strong auto-absorption of the Al resonance lines (394.4 and 396.15 nm), and spectral overlap with common alloying elements, such as Cu, Zn, and Mg. However, the authors demonstrated that advanced regression strategies can significantly improve LIBS quantification by reducing prediction errors across multiple alloying elements. Wang et al.^30^ applied a backpropagation artificial neural network (BP-ANN) to LIBS analysis of Al-Mg alloys. The authors demonstrated that nonlinear modeling of spectral features outperforms conventional calibration methods by capturing the complex relationships between plasma emission and alloy composition. In a recent study, ML was applied to LIBS of five aluminum alloy classes. Nonlinear reduction methods were compared with linear alternatives, such as principal component analysis (PCA), to mitigate the effects of high dimensionality and nonlinear relationships. The results showed that combining isometric mapping (IsoMap) nonlinear reduction with a support vector machine achieved 96.67% accuracy^31^.

The demand for rapid, accurate, and cost-effective characterization of aluminum alloys is increasing in sectors such as aerospace, automotive, and recycling, where precise control of alloy composition is critical for performance and sustainability. LIBS offers unique advantages for on-site analysis, yet its quantitative reliability is compromised by matrix effects, self-absorption, and spectral overlaps inherent to aluminum systems. Machine learning provides a pathway to overcome these limitations by leveraging the full spectral information to predict elemental concentrations without exhaustive plasma diagnostics. In this study, we comparatively evaluate three tree-based regression models—RF, GB, and ET—using a comprehensive LIBS dataset of aluminum and its alloys. Our goal is to explore the potential of these models for accurate and robust quantitative analysis, providing an effective alternative to traditional calibration strategies and deep learning approaches in metallurgical applications.

**Table 1:** **–** Comparison between tree-based models for LIBS elemental quantification.

Model	Key Characteristics	LIBS Applications
Random Forest (RF)	- Enables evaluation of each feature to the prediction. - Robust to noise and overfitting. - Can be less accurate in capturing nonlinear patterns.	^23^ ^24^ ^32^
Gradient Boosting (GB)	- Well-suited for complex nonlinear relationships. - Requires careful tuning of hyperparameters. - More prone to overfitting for noisy data.	^25^ ^26^
Extremely Randomized Trees (ET)	- Introduces extra randomness by split thresholds at random. - Performs well on noisy or redundant data. - May introduce higher bias due to random splits.	^33^

## Materials and methods

### Materials

Commercially pure aluminum (cp-Al) and aluminum alloys (Al-Cu, Al-Zn, and Al-Cu-Zn) Certified Reference Materials (CRM) with documented elemental compositions were used for the parallel training of multiple models (RF, GB, and ET) and their validation as regression modeling of LIBS data. Table 2 reports the chemical elements and their weight percentages (wt%) of these samples, certified by MBH Analytical Limited.

**Table 2:** Chemical composition of the samples of cp-Al and its alloys.

Nomenclature	Chemical Composition (wt%)					
	Reference Samples (for training of the models)					
	Al**	Cu	Zn	Mg	Fe	Si
cp-Al-67990*	98.741	0.074±0.002	0.140±0.010	0.054±0.002	0.670±0.020	0.322±0.011
cp-Al-11697	99.626	0.022±0.001	0.042±0.002	0.010±0.001	0.200±0.005	0.100±0.005
cp-Al-11628	97.910	0.150±0.022	0.197±0.012	0.118±0.005	0.815±0.022	0.810±0.032
Al-Cu E113	94.274	4.030±0.020	0.236±0.002	0.481±0.007	0.427±0.003	0.552±0.004
Al-Cu E115	95.699	0.658±0.010	0.051±0.002	0.922±0.000	1.080±0.000	1.590±0.020
Al-Cu E116*	92.566	6.610±0.030	0.566±0.000	0.035±0.001	0.122±0.003	0.101±0.002
Al-Zn G77J5	89.968	0.122	7.570	0.720	1.320	0.300
Al-Cu-Zn G77J1	90.490	2.410	1.910	4.830	0.210	0.150
Al-Cu-Zn G77J2*	90.871	2.370±0.040	3.250±0.050	3.040±0.050	0.293±0.005	0.206±0.007
Al-Cu-Zn G77J6	84.546	1.130±0.030	11.60±0.15	2.630±0.040	0.054±0.007	0.040±0.010
	Predicted Samples (for validation of the models)					
cp-Al-11694	99.388	0.006±0.002	0.035±0.002	0.002±0.005	0.430±0.010	0.140±0.005
cp-Al-11695	99.272	0.040±0.001	0.074±0.004	0.074±0.001	0.370±0.010	0.170±0.010
Al-Cu E114	98.873	5.470±0.05	0.125±0.003	0.108±0.000	0.188±0.005	0.236±0.004
Al-Cu-Zn G77J4	90.790	0.810±0.020	5.300±0.060	1.470±0.030	1.040±0.030	0.590±0.020

*Samples used for both training and in-sample (non-independent) validation of the models.**The Al content was obtained by difference (100 – Σ other elements), as specified in the MBH Analytical Limited certificates.

The CRM surfaces were prepared for LIBS testing by sequential sanding from #220 to #1200 grit sandpaper, followed by cleaning with ethanol and drying.

### Experimental setup of LIBS

**Fig. 1:**
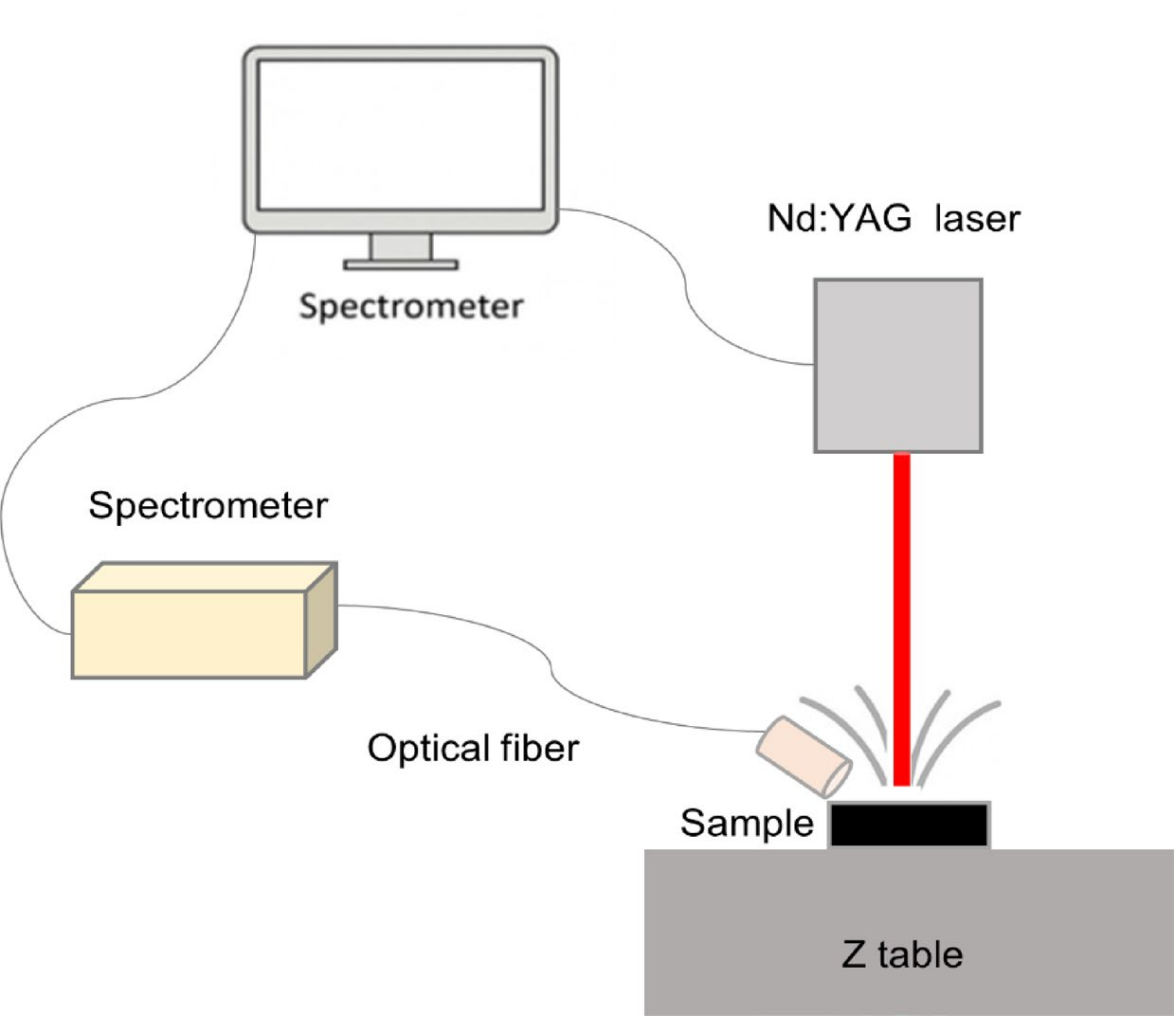
Schematic diagram of LIBS experimental setup.

Figure 1 shows the experimental setup of laser-induced breakdown spectroscopy (LIBS), which was designed and assembled at CENIM-CSIC. For all LIBS measurements in this study, plasma was generated using a Q-switched Nd: YAG laser (Onteko) with a wavelength of 1064 nm, a pulse duration of 6 ns, a repetition rate of 1 Hz and a pulse energy of 10 mJ. During the process, the laser beam is focused on the sample surface at a focal distance of 50 mm to produce a plasma plume. A photodetector captured the light emitted by the plasma with optimized delay times for each alloy system to maximize the signal-to-background ratio (SNR) within the 250–850 nm range. For cp-Al and Al-Cu alloys, a delay time of 5 µs was sufficient since the Al and Cu lines are intense at the early stages of plasma emission and provide a high SNR. However, a longer delay time of 15 µs was required for Al–Zn and Al–Cu–Zn alloys to avoid detector saturation observed at shorter delays (5 µs) and to improve the detection of Zn emission lines. At this later stage of plasma evolution, the continuum background decreases and the relative line intensities become more distinguishable, enhancing line resolution and signal-to-background ratio. This behavior is consistent with the temporal evolution of LIBS plasmas, in which emission characteristics depend strongly on time due to changes in plasma temperature, electron density, and continuum radiation^3,4^.

The emitted light is collected by an optical fiber and directed to a spectrometer (Ocean Optics USB4000), which disperses the light and records the spectral data with acquisition software (SpectraGryph 1.2).

In the present study, each CRM sample was analyzed at 50 different positions, with 10 consecutive laser pulses applied at each position. Only the emission spectrum from the tenth pulse was recorded because the initial pulses were used primarily to clean the surface and stabilize plasma formation. Laser parameters and detection delay times were selected based on preliminary tests and optimization procedures to ensure acquisition of spectra with the highest signal-to-background ratio (SNR).

### Quantification of chemical elements from LIBS data using RF, GB, and ET models

A total of 500 spectra, obtained from ten CRM aluminum samples with varying concentrations of aluminum (Al), copper (Cu), zinc (Zn), magnesium (Mg), iron (Fe), and silicon (Si), were used to train three tree-based models: RF, GB, and ET. The machine learning analysis was implemented in Python 3.12^34^ using Google Colab^35^, a cloud-based Jupyter notebook environment running Ubuntu 22.04 LTS. All computations were performed on a Colab virtual machine without GPU acceleration. Details of the scripts are described in the following sections.

#### Data acquisition and preprocessing of LIBS spectra

Initially, LIBS measurements were performed by applying ten consecutive laser pulses at each analysis position. Only the emission spectrum from the tenth pulse was recorded and saved as an individual CSV file containing paired wavelength-intensity data. Thus, each analysis position yielded one representative spectrum while the first nine pulses cleaned the surface and stabilized the plasma. For each sample, 50 positions were analyzed, resulting in 50 spectra per sample. Considering ten different reference samples, a total of 500 spectra were obtained. Prior to modeling, the spectra underwent a multi-step preprocessing workflow. The high-frequency noise was reduced, and peak definition was enhanced by applying a *Savitzky–Golay* filter^36^ with a fixed window size of 17 and a polynomial order of 2. The evaluated spectral range was 250–850 nm. The *Savitzky-Golay* filter parameters were selected as a compromise between noise reduction and preservation of emission-line features. These parameters were defined based on preliminary tests evaluating different configurations to ensure consistent peak definition across the analyzed spectral range. Spectral intensities were then interpolated at 0.1 nm intervals and normalized to their maximum intensity to ensure comparability across samples. No feature selection or peak extraction was performed. Instead, the full preprocessed spectrum was used as input for the machine learning models, consisting of interpolated intensity values across the entire 250–850 nm range (~ 6,000 variables). This approach allows the models to capture both strong emission lines and subtle spectral features associated with minor and trace elements.

Finally, a complete dataset was constructed by combining the interpolated and normalized spectral intensities (model features) with the corresponding certified elemental compositions (targets). All processed spectra were consolidated into a single dataset and exported as a CSV file for training and validating a machine learning model.

#### Regression modeling of LIBS data for multi-element analysis

Three regression models based on machine learning methods were trained and evaluated to quantify the elemental composition from LIBS spectra: RF, GB, and ET. RF employs multiple decision trees, each built from a randomly sampled subset of features. The individual strength of the trees and their mutual correlation result in a generalization error that tends to converge^33^. GB builds predictive models that optimize a loss function using regression trees as base learners. This enables predictions for both regression and classification tasks, even with noisy data^37^. ET is a method designed for classification and regression tasks. It employs strong randomization to generate highly diversified trees with computational efficiency and robustness while maintaining competitive predictive accuracy^38^. These models were implemented using the *scikit-learn library*^39^, which provides practical tools for developing, training, evaluating, and deploying machine learning models in Python.

The previously generated dataset containing interpolated spectral intensities as input features and corresponding real elemental compositions as target variables was used for model training. The data were randomly split into training (80%) and test (20%) subsets at the spectrum level using a fixed random seed (42). In this approach, spectra from the same sample could be present in both subsets. This strategy was adopted to evaluate model performance at the spectral level, which is representative of typical LIBS measurements involving multiple acquisitions per sample. To assess model generalization and mitigate potential bias associated with this splitting strategy, external validation was performed using independent samples not included in the training dataset, as described in Sect. 2.3.3. This complementary validation ensures that the performance of the model is not overestimated because of the spectrum-level splitting strategy.

The RF and ET models were trained directly. In contrast, the GB model was wrapped in a MultiOutputRegressor to handle multiple outputs simultaneously. Each model was trained using 100 to 400 estimators (trees), and the optimal number of estimators was selected based on the minimization of the Mean Squared Error (MSE) on the test subset. This strategy contributes to controlling model complexity, reducing the risk of overfitting and underfitting during training, while the use of ensemble tree-based models (RF, GB, and ET) provides inherent robustness when handling high-dimensional spectral data. When similar predictive performances were observed across different configurations, the model with lower computational cost (i.e., fewer estimators and reduced training time) was preferred. Training and evaluation of the models were conducted in parallel using the joblib library to optimize computational performance. Model performance was evaluated using element-wise Mean Square Error (MSE) and Root Mean Squared Error (RMSE)^40^, while the coefficient of determination (R²)^41^ was used as a global metric at the sample level. Finally, the trained models were saved individually in pickle format for further use in prediction tasks.

#### Application of trained models to new LIBS spectra

To estimate the chemical composition of the reference and unknown (predicted) samples (Table 2), the trained models (RF, GB, and ET) were applied to the LIBS spectra collected from these samples. Each input spectrum was preprocessed with smoothing, wavelength filtering, interpolation, and normalization to generate input features, as previously described in Sect. 2.3.1. The three models were saved in pickle format and loaded using the *joblib library* to predict the elemental concentrations in weight% (wt%). Reference samples were used to evaluate the behavior of the models and its consistency, while predicted samples were used to assess the model ability to predict the composition of the new samples.

The predictions for each model were compiled into structured data frames, and the mean composition of each element was calculated across all analyzed spectra (50 files per sample). The results were exported as bar graphs and an Excel file was generated with individual sheets containing the predictions of each model.

The agreement between predicted and reference values was assessed using student t-tests (α = 0.05). The analysis considered both the variability of the predicted means (standard deviation of replicate spectra) and the reported uncertainties of the certified reference values, when available. This approach follows standard statistical procedures for evaluating the accuracy and uncertainty of measurement data^42,43^. The student t-test was not performed on aluminum (Al), since its reference values were obtained by difference (100 wt% – sum of other alloy elements) and thus lacked an experimentally measured standard deviation.

Model validation was carried out at two levels: (i) spectrum-level validation, which involved 80/20 random split across 500 spectra, and (ii) sample-level validation, which entailed aggregating spectra from individual alloys. Two scenarios were considered at the sample level: in-sample (non-independent) validation, in which the ten samples were included in the training dataset, and external validation, in which the independent samples E114, G77J4, 11,694, and 11,695 were not used for training. In-sample validation was used to evaluate the physical and chemical consistency of the models at the sample scale. It is important to highlight that this validation is not independent, since spectra from the same samples may appear in both the training and test subsets due to the spectrum-level data split. Therefore, this analysis assesses the internal consistency of the models rather than their generalization capability.

Additionally, the model performance was evaluated using standard regression metrics: mean squared error (MSE), mean absolute error (MAE), root mean squared error (RMSE)^44^, and the coefficient of determination (R²). MSE and RMSE emphasize larger deviations by penalizing squared errors. In contrast, MAE provides a more direct measure of average prediction error in weight% (wt%). R² quantifies the proportion of the variance in the reference composition explained by the model; values closer to 1 indicate higher predictive accuracy. In the context of LIBS analysis, where plasma fluctuations, matrix effects, and spectral overlap introduce significant variability, these metrics allow us to assess both the magnitude of prediction errors (MAE and RMSE) and the overall model fit (MSE and R²). Together, these metrics provide a framework for evaluating the predictive reliability of regression models applied to spectroscopic data.

The full pipeline, including preprocessing and training scripts, is archived at Zenodo^45^.

## Results and discussion

### Internal spectrum-level validation of ensemble regression models

**Fig. 2:**
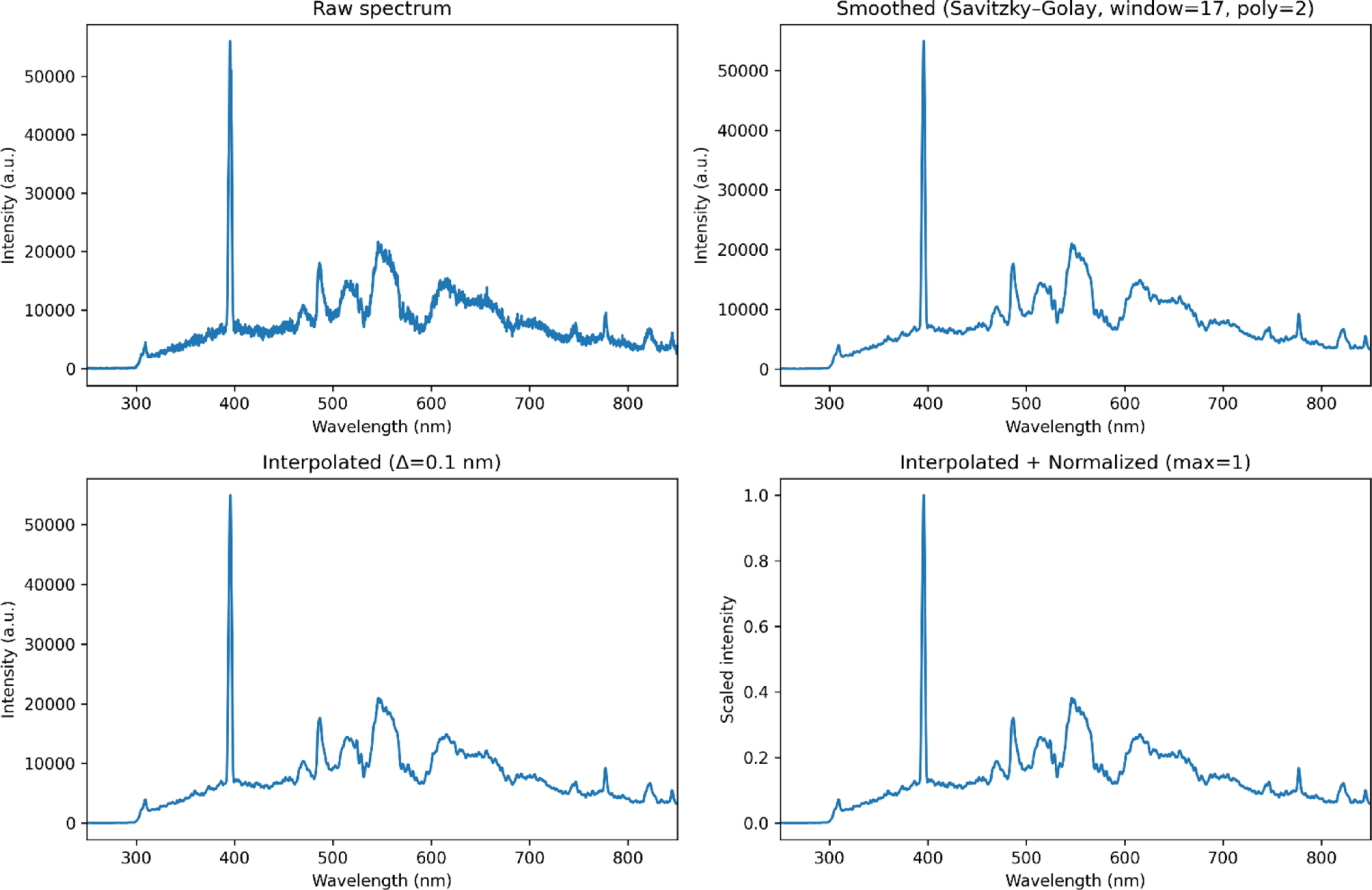
Example of the LIBS pre-processing workflow applied to a representative spectrum: (**a**) raw spectrum, (**b**) *Savitzky–Golay* smoothing, (**c**) interpolation at 0.1 nm, and (**d**) max normalization.

Figure 2 illustrates the preprocessing workflow applied before model training. It shows a representative LIBS spectrum of a cp-Al sample at four stages: raw acquisition, after *Savitzky–Golay* smoothing, interpolated at 0.1 nm, and normalized to maximum intensity. This visualization clarifies how spectral features were standardized before machine learning analysis. An advantage of this workflow is that it does not require baseline subtraction or background smoothing. Combining *Savitzky–Golay* filtering and normalization reduced high-frequency noise, standardized the spectra, and preserved emission-line intensities. This simplified the preprocessing pipeline and avoided the risk of introducing distortions caused by baseline correction methods.

Table 3 presents the performance of the models, which is based on an internal 80/20 split of the dataset. The dataset comprises 500 LIBS spectra from ten reference samples with varying chemical compositions (see Table 2). The evaluation was conducted at the spectrum level, meaning spectra from the same sample could appear in both the training and test subsets. RF model achieved an MSE of 0.1548 and an R² of 0.9517. GB model improved the fit, achieving an MSE of 0.0837 and an R² of 0.9715. ET model reduced the error further, achieving an MSE of 0.0429 and an R² of 0.9880. These results demonstrate that the ET model outperformed the other algorithms by providing the lowest error and the highest coefficient of determination. This superior performance becomes clearer when considering the nature of LIBS data and the limitations of conventional linear approaches. For example, although Partial Least Squares (PLS) regression is widely used in LIBS quantitative analysis due to its simplicity and interpretability, it assumes linear relationships between spectral intensities and elemental concentrations. PLS models typically provide comparable performance under controlled conditions and are limited when dealing with nonlinear behavior and compositional complexity^46,47^. Studies have shown that machine learning approaches can outperform traditional linear methods in LIBS quantitative analysis with higher R² values and lower prediction errors^48,49^. As previously mentioned, the literature highlights these limitations, demonstrating that deviations from linearity are prevalent due to plasma-matter interactions and self-absorption effects^1,50^.

The ET model enhanced performance can be attributed to its greater randomness in feature and threshold selection during tree construction. Additionally, the ET algorithm is more robust to noise and outliers because of its randomized splitting strategy. It is also less sensitive to feature scaling because tree-based methods rely on threshold-based decisions rather than distance metrics. These characteristics contribute to the ET algorithm stability when handling high-dimensional, noisy LIBS spectral data These properties are well documented for ensemble tree methods and contribute to their effectiveness in high-dimensional and noisy datasets^38^. This is particularly advantageous for LIBS data analysis because plasma fluctuations, matrix effects, and background noise introduce nonlinearities. Consequently, the ET model can achieve a more effective balance between bias and variance, resulting in improved generalization performance. Similar findings were reported by Geurts et al.^38^, who demonstrated that adding randomness to the selection process reduces generalization error in classification and regression tasks.

As previously mentioned, LIBS analysis of aluminum and its alloys presents several challenges. These include high thermal conductivity, low ablation thresholds, and the pronounced self-absorption of Al resonance lines at 394.4 and 396.15 nm. There is also spectral overlap with alloying elements such as copper, zinc, and magnesium^3,4,29^. These effects reduce reproducibility, making it more difficult to extract reliable features for quantitative analysis. The superior performance of ET algorithms in this study suggests that they are well-suited for LIBS applications involving aluminum alloys, as they can handle nonlinearities and noise, which often causes conventional calibration strategies to fail to compensate for matrix effects. By incorporating greater randomness in tree construction, the ET model can capture subtle spectral variations associated with minor alloying elements. This mitigates distortions introduced by plasma instabilities and line self-absorption.

**Table 3:** Performance of three trained models for 500 randomly selected LIBS spectra.

Trained model	MSE	*R* ^2^
RF	0.1548	0.9517
GB	0.0837	0.9715
ET	0.0429	0.9880

Increasing the number of estimators for RF and ET models from 100 to 400 did not result in significant improvements in predictive accuracy. This confirms that merely expanding the ensemble size does not guarantee better performance in LIBS modeling. Similar conclusions were reported by Friedman et al.^51^, who emphasized that inappropriate hyperparameter tuning, such as a suboptimal learning rate, maximum tree depth, or number of iterations, can result in overfitting or underfitting.

Processing the LIBS-ML training took about 68 min for 500 spectra. This behavior can be attributed to the cost of fitting three ensemble models—RF and ET, each with 100 trees, and GB, with 400 trees – using approximately 6000 input features (derived from the 0.1 nm interpolation of each spectrum), within the computational environment of a Google Colab virtual machine.

### Validation of regression models by sample

In addition to the internal, spectrum-level validation presented in Table 3, the model performance at the sample level was examined under two scenarios: semi-external (samples used in the training set) and external (samples E114, G77J4, 11694, and 11695). These analyses, together, provide insight into the consistency and generalization ability of the ensemble regression models.

#### In-sample (non-independent) validation: samples included in the training dataset

**Fig. 3:**
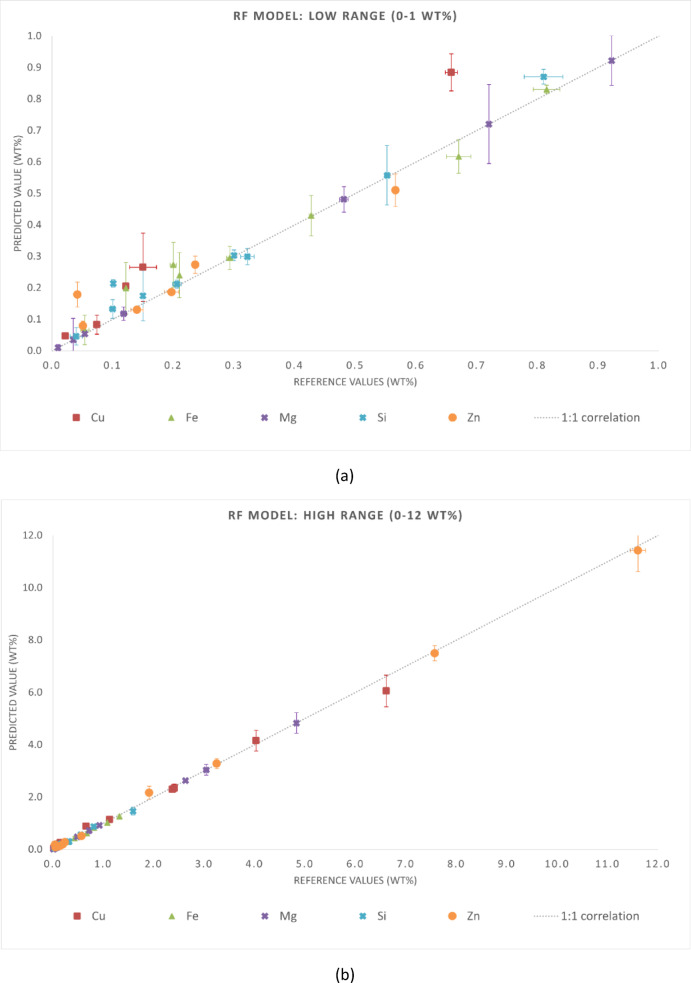
Reference versus predicted compositions for the RF model using in-sample (non-independent) predictions. Axis breaks highlight the ranges (**a**) 0–1 wt% and (**b**) 1–12 wt%. The dashed line represents the 1:1 correlation.

**Fig. 4:**
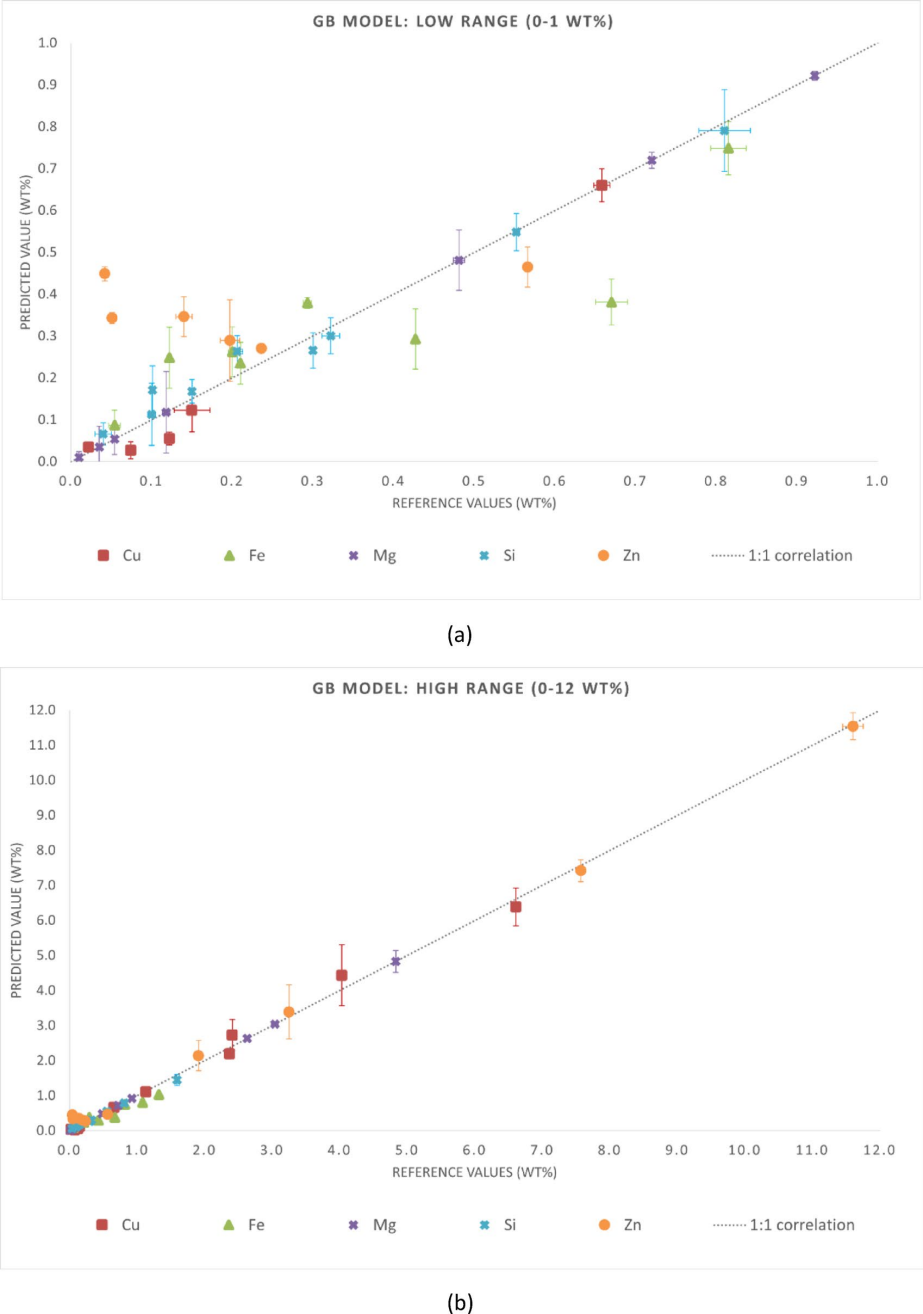
Reference versus predicted compositions for the GB model using in-sample (non-independent) predictions. Axis breaks highlight the ranges (**a**) 0–1 wt% and (**b**) 1–12 wt%. The dashed line represents the 1:1 correlation.

**Fig. 5:**
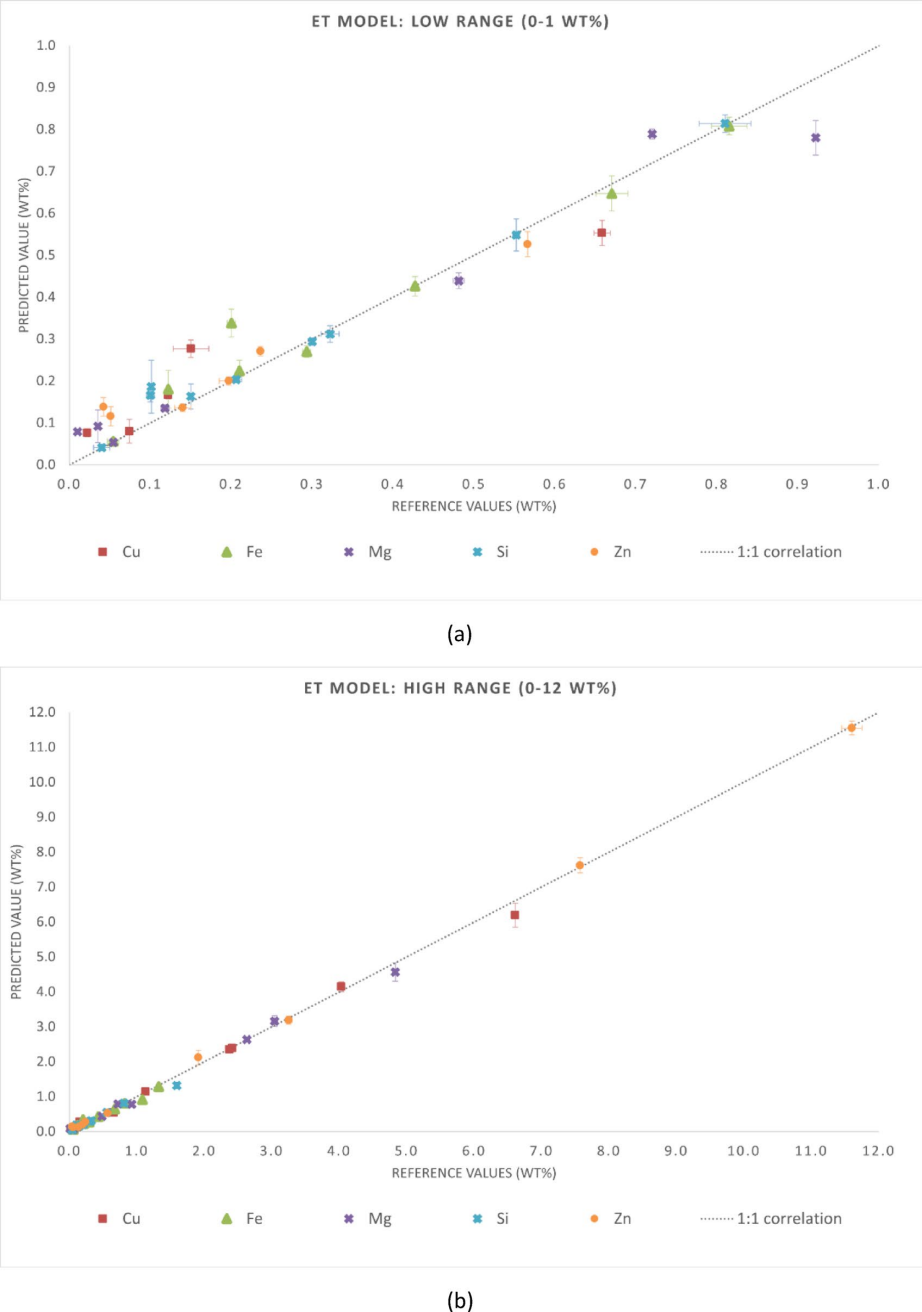
Reference versus predicted compositions for the ET model using in-sample (non-independent) predictions. Axis breaks highlight the ranges (**a**) 0–1 wt% and (**b**) 1–12 wt%. The dashed line represents the 1:1 correlation.

The statistical metrics of the predictive performance of the three regression models are presented in Tables 4 and 5. Table 4 reports MAE and RMSE for each chemical element, while Table 5 summarizes the global metrics (MAE, RMSE, and R²) for the entire dataset. These results align with the graphical trends observed in Figs. 3, 4 and 5. Although this is not independent validation, evaluating the samples in the training set provides insight into the consistency of predictions when spectra are aggregated at the sample level. It is important to note that this analysis is not independent validation. Due to the spectrum-level data splitting strategy adopted in this study, spectra from the same samples were included in both the training and test subsets. Thus, the evaluation in this section is an in-sample (non-independent) validation that reflects the model consistency in predicting spectra from samples already in the training dataset. This analysis assessed whether the models preserve the chemical identity of each alloy when multiple spectra are averaged, which is representative of practical LIBS applications.

For aluminum-rich samples (cp-Al), all models exhibited high accuracy. Predictions were closely aligned with reference values and showed minimal dispersion. This behavior was observed across all three cp-Al samples (67990, 11697, and 11628), with deviations below 0.5 wt% for the quantified elements. The GB model showed higher scatter in this case, while the ET model provided the most accurate fits consistently. The RF model exhibited intermediate performance, closely following the ET model for most cp-Al predictions. For the Al–Cu alloy (samples E113, 115, and E116), all models slightly underpredicted Cu. This tendency was more pronounced in sample E116, where 6.6 wt% Cu was underestimated by all algorithms, especially the RF and GB models. For the Al–Cu–Zn ternary alloy (sample G77J2), the ET model most closely agreed with the Zn reference values. ET model reproduced both intermediate Zn levels (3.25 wt% in sample G77J2) and high Zn content (11.60 wt% in sample G77J6) with minimal bias. In contrast, the RF and GB models exhibited larger deviations at the highest Zn concentrations. For the cp-Al sample (67990), where trace elements dominate the residuals, the ET model outperformed the others, while the GB model displayed the greatest deviation. These trends were confirmed by the predicted versus reference plots. Aluminum-rich matrices exhibited strong linearity across all algorithms, whereas secondary alloying elements showed slight but systematic deviations. Linearity was preserved for major and secondary elements up to 12 wt% across all ten samples, confirming the robustness of the models over a wide compositional range. Prediction accuracy decreased for all models when considering minor constituents (less than 1 wt%), with increased scatter around the 1:1 line. This behavior reflects the known challenges of LIBS at trace levels, including plasma fluctuations, spectral overlap, and matrix effects. The additional randomness of the ET model reduced systematic bias and improved generalization, thereby enhancing its performance. Meanwhile, RF remained intermediate between GB and ET.

The results demonstrated that all three models can accurately predict the major and secondary constituents of cp-Al and Al-based alloys when evaluated under non-independent conditions. Among the tested approaches, the ET model appears to be the most reliable regressor, providing the most consistent predictions without requiring extensive hyperparameter tuning.

#### External validation: independent samples

**Fig. 6:**
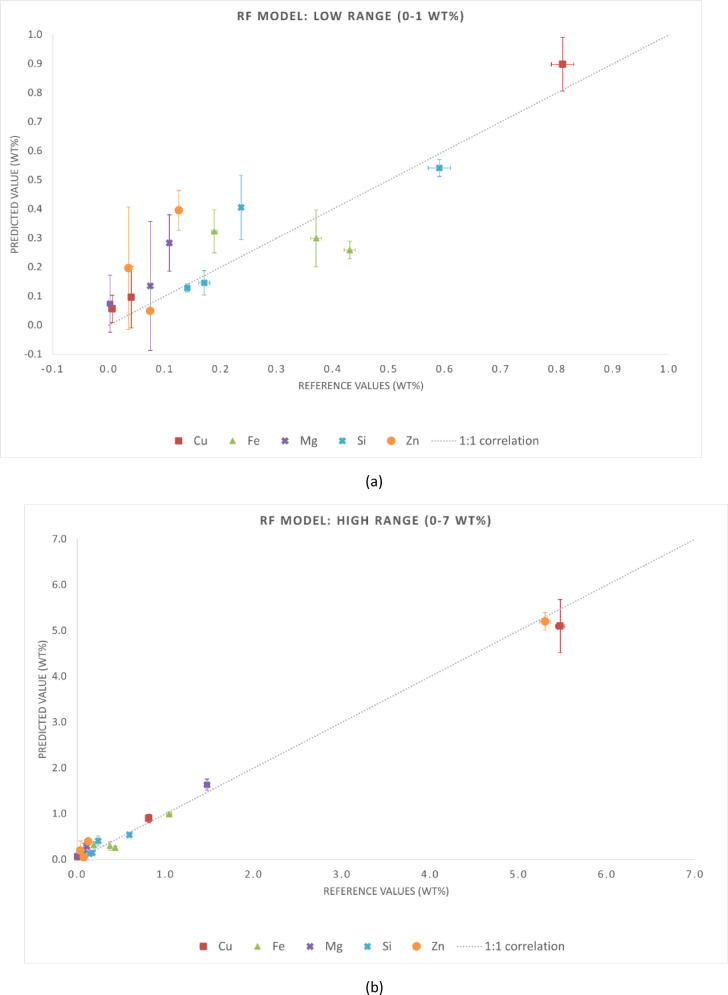
Reference versus predicted compositions obtained with the RF model for the validation samples (E114, G77J4, cp-Al 11694, and cp-Al 11695). Axis breaks highlight the ranges (a) 0–1 wt% and (b) 1–7 wt%. The dashed line represents the 1:1 correlation.

**Fig. 7:**
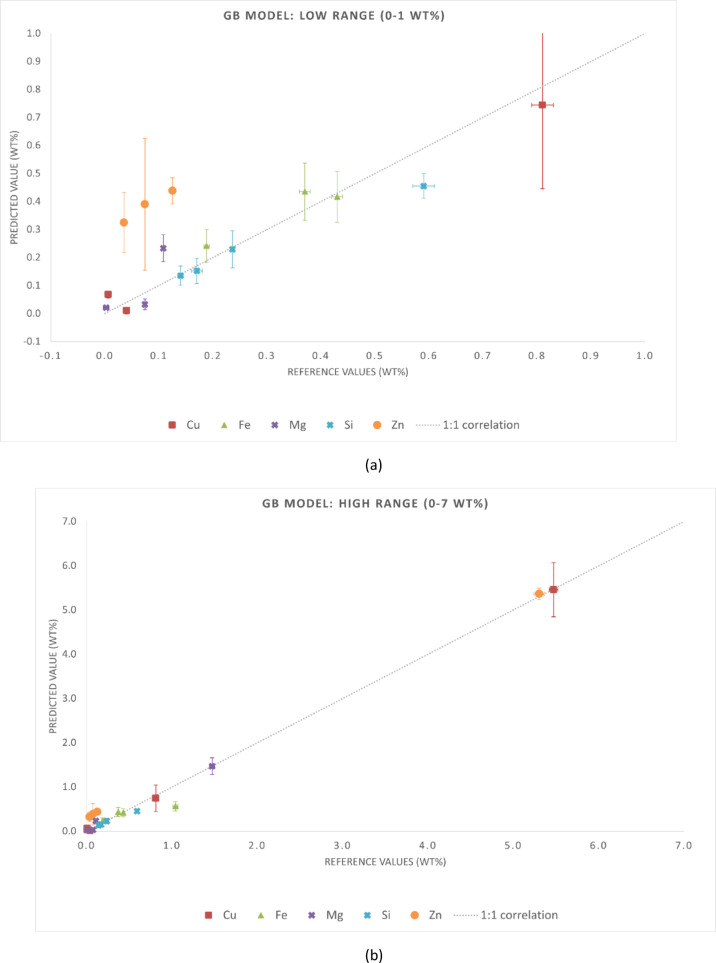
Reference versus predicted compositions obtained with the GB model for the validation samples (E114, G77J4, cp-Al 11694, and cp-Al 11695). Axis breaks highlight the ranges (**a**) 0–1 wt% and (**b**) 1–7 wt%. The dashed line represents the 1:1 correlation.

**Fig. 8:**
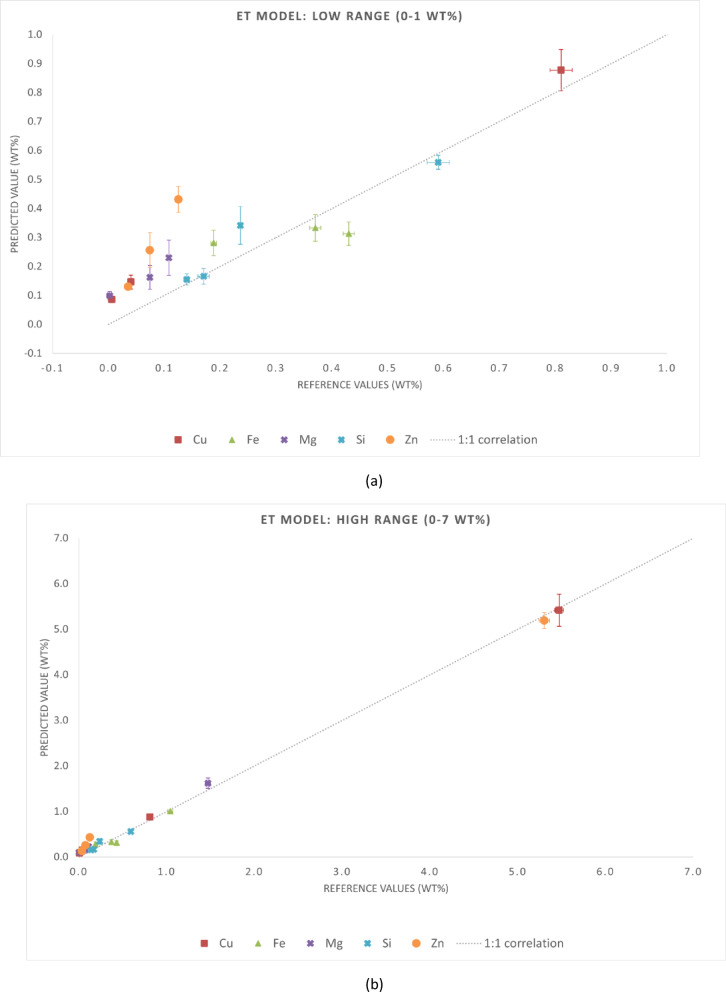
Reference versus predicted compositions obtained with the ET model for the validation samples (E114, G77J4, cp-Al 11694, and cp-Al 11695). Axis breaks highlight the ranges (**a**) 0–1 wt% and (**b**) 1–7 wt%. The dashed line represents the 1:1 correlation.

The predictive performance of the regression models was also assessed using independent LIBS spectra from validation samples not included in the training set (Table 2). Figures 6, 7, 8 compare the reference and predicted compositions obtained with the three models.

The results indicate that the three ensemble regression models (RF, GB, and ET) accurately captured the overall compositional trends of the validation samples. In the binary Al-Cu alloy (sample E114), copper (Cu) was correctly identified as the secondary element at 5–6 wt% while minor constituents (Fe, Mg, Si, and Zn) were detected at trace levels of less than 0.5 wt%. However, their predictions displayed slight, model-dependent fluctuations. Although the absolute concentrations were correctly identified, the predictions showed dispersion depending on the element and model, especially in the low-concentration range. For the ternary Al-Cu-Zn alloy (sample G77J4), the models correctly identified Cu (approximately 1 wt%) and Zn (5–6 wt%) as relevant alloying elements. Predictions for Fe, Mg, and Si remained below 2 wt%, consistent with their expected minor role. ET model showed the closest agreement with the reference values for Zn, while GB model displayed slightly higher scatter in this range. The three models also maintained high predictive accuracy for commercially pure aluminum (samples 11694 and 11695). Residual trace elements (Fe, Mg, Si, and Zn) were predicted at levels below 2 wt%, reflecting their minor role. The differences between the models were generally subtle for major elements, whereas clearer discrepancies emerged for trace constituents, particularly in the low-concentration regime. In this regime, ET showed greater robustness, while GB exhibited higher sensitivity to spectral noise. The slightly higher prediction errors observed for Al-Cu and Al-Cu-Zn alloys (samples E114 and G77J4, respectively) are influenced by alloy type and compositional complexity. Binary and especially ternary alloys involve more complex multi-element interactions, making prediction more difficult. Additionally, the presence of multiple alloying elements and trace constituents increases spectral complexity and reduces the signal-to-noise ratio of minor elements, further contributing to the observed increase in prediction error.

It is important to note that Zn exhibited more pronounced deviations in the external validation samples (Figs. 6, 7, 8). This behavior is not observed in in-sample (non-independent) validation, where Zn predictions, particularly for the ET model, are more accurate. These findings suggest that the observed deviations can primarily be attributed to low Zn concentration levels (trace regime) and increased matrix complexity in ternary alloys, such as G77J4.

A statistical comparison based on t-tests revealed that the GB model produced the greatest number of cases in which the predictions were statistically indistinguishable from the reference values within the experimental uncertainty (7 out of 24 cases, *p* > 0.05). The ET and RF models followed with four and two such cases, respectively. While this suggests that GB predictions more often overlap with reference values, it is largely a consequence of its higher prediction variances. These variances inflate the standard errors and consequently the p-values, masking systematic deviations. In contrast, the ET model exhibited statistical differences from the reference values in most cases. However, this was due to its lower dispersion, which made the test more sensitive to small systematic biases. A complete list of p-values for each sample and element is provided in Supplementary Table S1. This behavior suggests that, although the GB model appears statistically consistent with the reference values, its higher prediction variance makes it unreliable for precise quantitative applications. In LIBS analysis, where the accurate estimation of both major and trace elements is critical, lower dispersion is generally preferred to statistical indistinguishability driven by uncertainty. Therefore, the ET model provides more consistent and reliable predictions for practical compositional analysis, despite the higher p-values observed for GB model.

**Fig. 9:**
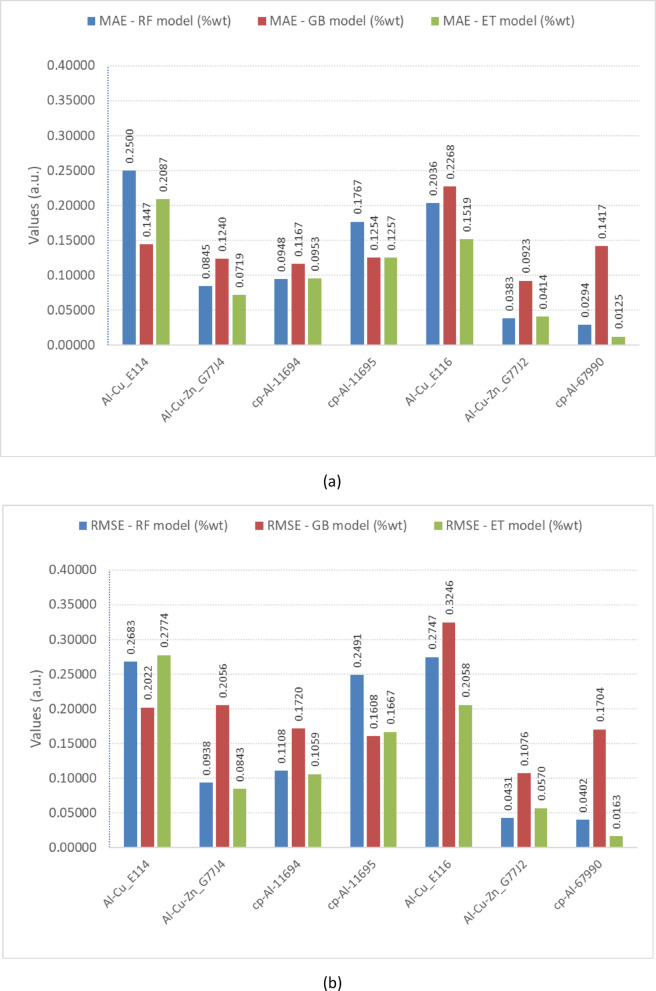
Comparison of prediction errors for the validation samples: (**a**) mean absolute error (MAE) and (**b**) root mean squared error (RMSE) obtained for the RF, GB, and ET models.

To integrate the results of the semi-external and external validations, we assessed the global predictive performance using the mean absolute error (MAE), the root mean squared error (RMSE), and the coefficient of determination (R²). Figure 9 shows the MAE (Fig. 9a) and RMSE (Fig. 9b) values for all models (RF, GB, and ET) across the validation samples. Although not shown in Fig. 9, the R² values remained consistently close to unity for all models, indicating a high level of predictive accuracy. Overall, the three ensemble models (RF, GB, and ET) produced low error metrics, with MAE values generally below 0.25 wt% and RMSE values below 0.33 wt%. In LIBS, jointly analyzing these metrics is particularly relevant because MAE and RMSE quantify absolute and squared deviations, respectively, capturing errors at major and trace levels.

For the Al-Cu alloys (samples E114 and E116), the ET model generally outperformed the RF and GB models. It achieved the lowest error in sample E116 (MAE = 0.1519%; RMSE = 0.2058%). For the ternary alloys (samples G77J2 and G77J4), the ET model again provided the most accurate results, while the GB model showed higher deviations. The ET model also yielded the best predictions for cp-Al (sample 67990: MAE = 0.0125 wt%; RMSE = 0.0163 wt%), substantially reducing errors compared to the RF and GB models.

Finally, when comparing semi-external and external validation, error metrics remained consistently low in both sets. For the semi-external samples, ET model achieved an MAE of 0.0125 wt% (cp-Al 67990) and RMSE values below 0.21 wt% for the alloys. For the external samples, the MAE remained in the same range (< 0.25 wt%), and the RMSE values were below 0.33 wt%. This indicates that transitioning from training-pool samples to independent ones did not significantly increase predictive error. Instead, the models—particularly ET model —preserved high accuracy and generalization capacity, confirming that they were learning underlying relationships rather than memorizing the training set. This consistency across metrics reinforces that the ensemble regressors were effectively generalizing from LIBS training data, capturing real compositional relationships rather than memorizing spectra. Accordingly, the high R² values observed are not indicative of overfitting but reflect the ability of the models to generalize to independent samples not included in the training dataset.

**Table 4:** Predictive performance of the three regression models (RF, GB, and ET) based on MAE and RMSE for each chemical element.

Sample	Metric %wt	Al			Cu			Fe			Mg			Si			Zn		
		**RF**	**GB**	**ET**	**RF**	**GB**	**ET**	**RF**	**GB**	**ET**	**RF**	**GB**	**ET**	**RF**	**GB**	**ET**	**RF**	**GB**	**ET**
cp-Al-67990	MAE	0.0870	0.2098	0.0447	0.0184	0.1332	0.0114	0.0527	0.2887	0.0225	0.0046	0.0489	0.0031	0.0239	0.0381	0.0104	0.0095	0.2062	0.0047
	RMSE	0.1347	0.2731	0.0906	0.0349	0.2070	0.0284	0.0747	0.2937	0.0471	0.0073	0.0567	0.0078	0.0348	0.0477	0.0222	0.0138	0.2115	0.0099
cp-Al-11697	MAE	0.3068	0.9329	0.4235	0.0251	0.0763	0.0536	0.0251	0.0731	0.1383	0.0389	0.0730	0.0692	0.0331	0.0383	0.0663	0.1366	0.4068	0.0961
	RMSE	0.6877	1.0956	0.6377	0.0534	0.1137	0.1074	0.0534	0.1017	0.1421	0.1085	0.1549	0.1744	0.0443	0.0747	0.0681	0.4168	0.4407	0.2379
cp-Al-11628	MAE	0.2526	0.3699	0.1432	0.1153	0.0455	0.1274	0.0174	0.0669	0.0117	0.0719	0.1907	0.0167	0.0604	0.0529	0.0146	0.0107	0.0924	0.0058
	RMSE	0.2766	0.4421	0.2302	0.1577	0.0580	0.2431	0.0201	0.0913	0.0224	0.0749	0.2135	0.0184	0.0648	0.0983	0.0208	0.0116	0.1334	0.0107
Al-Cu_E113	MAE	0.2041	0.6208	0.1256	0.2788	0.8244	0.1531	0.0323	0.1426	0.0165	0.0491	0.0653	0.0437	0.0464	0.1180	0.0283	0.0405	0.2396	0.0348
	RMSE	0.2573	0.6500	0.1446	0.4071	0.9504	0.1826	0.0634	0.1519	0.0229	0.0573	0.0777	0.0462	0.0938	0.1308	0.0376	0.0460	0.2643	0.0363
Al-Cu_E115	MAE	0.1834	0.5580	0.3814	0.2434	0.1633	0.0895	0.0642	0.2776	0.8872	0.0905	0.0786	0.1416	0.1254	0.1434	0.2706	0.0281	0.2922	0.0654
	RMSE	0.3112	0.6012	0.4125	0.6243	0.3883	0.0944	0.1168	0.2925	0.8886	0.1194	0.1116	0.1472	0.1964	0.2098	0.2812	0.0509	0.3201	0.0693
Al-Cu_E116	MAE	0.3411	0.7388	0.2563	0.5553	0.2512	0.4161	0.0766	0.1268	0.0578	0.0796	0.1083	0.0567	0.1135	0.0719	0.0850	0.0554	0.1013	0.0397
	RMSE	0.5194	0.8481	0.3410	0.8170	0.5797	0.5366	0.1115	0.1456	0.0732	0.1045	0.1174	0.0685	0.1606	0.0903	0.1056	0.0755	0.1112	0.0496
Al-Zn_G77J5	MAE	0.1319	0.1731	0.1310	0.0832	0.1460	0.0452	0.0559	0.2920	0.0400	0.1110	0.1445	0.0693	0.0126	0.0463	0.0076	0.2033	0.2172	0.1305
	RMSE	0.1839	0.2820	0.1857	0.1394	0.1661	0.1095	0.0817	0.2967	0.0698	0.1667	0.1972	0.1379	0.0171	0.0542	0.0108	0.2964	0.3471	0.2174
Al-Cu-Zn_G77J1	MAE	0.1060	0.1640	0.0660	0.0652	0.4285	0.0203	0.0298	0.0326	0.0138	0.3019	0.1843	0.2618	0.0250	0.0247	0.0132	0.2752	0.2729	0.2159
	RMSE	0.3871	0.2401	0.1471	0.1576	0.5400	0.0404	0.0769	0.0556	0.0286	0.4920	0.3594	0.3737	0.0828	0.0329	0.0327	0.3564	0.4839	0.2922
Al-Cu-Zn_G77J2	MAE	0.0440	0.0792	0.0387	0.0716	0.1981	0.0219	0.0338	0.1287	0.0092	0.1104	0.0346	0.1234	0.0088	0.0606	0.0038	0.1311	0.3332	0.0804
	RMSE	0.0584	0.1285	0.0537	0.0996	0.2294	0.0363	0.0488	0.1742	0.0156	0.2087	0.0554	0.1947	0.0146	0.0687	0.0059	0.1904	0.7808	0.1305
Al-Cu-Zn_G77J6	MAE	0.1511	0.0962	0.0388	0.0152	0.0533	0.0066	0.0120	0.0411	0.0018	0.0096	0.0265	0.0030	0.0058	0.0295	0.0012	0.1747	0.0761	0.0506
	RMSE	0.6835	0.2910	0.1486	0.0894	0.0776	0.0280	0.0478	0.0485	0.0064	0.0345	0.0381	0.0141	0.0282	0.0374	0.0042	0.8134	0.3872	0.1982

**Table 5:** Predictive performance of the three regression models (RF, GB, and ET) based on global statistical metrics (MAE, RMSE, and R²).

Sample	Metric	RF model	GB model	ET model
		**Value**		
cp-Al-67990	MAE (%wt)	0.0294	0.1325	0.0125
	RMSE (%wt)	0.0402	0.1664	0.0163
	R²	1.000000	0.999985	1.000000
cp-Al-11697	MAE (%wt)	0.1023	0.2483	0.1412
	RMSE (%wt)	0.1422	0.4171	0.1914
	R²	0.999999	0.999986	0.999999
cp-Al-11628	MAE (%wt)	0.0876	0.1276	0.0501
	RMSE (%wt)	0.1199	0.1767	0.0786
	R²	0.999999	0.999994	0.999998
Al-Cu_E113	MAE (%wt)	0.0556	0.2470	0.0506
	RMSE (%wt)	0.0750	0.3095	0.0679
	R²	0.999998	0.999967	0.999998
Al-Cu_E115	MAE (%wt)	0.0934	0.2142	0.1885
	RMSE (%wt)	0.1160	0.2779	0.2168
	R²	0.999989	0.999976	0.999992
Al-Cu_E116	MAE (%wt)	0.2036	0.2268	0.1519
	RMSE (%wt)	0.2747	0.3246	0.2058
	R²	0.999951	0.999983	0.999973
Al-Zn_G77J5	MAE (%wt)	0.065715	0.115174	0.054853
	RMSE (%wt)	0.073461	0.145719	0.064652
	R²	0.999996	0.999990	0.999999
Al-Cu-Zn_G77J1	MAE (%wt)	0.1224	0.1546	0.0940
	RMSE (%wt)	0.1663	0.1881	0.1381
	R²	0.999975	0.999977	0.999983
Al-Cu-Zn_G77J2	MAE (%wt)	0.0383	0.0923	0.0414
	RMSE (%wt)	0.0431	0.1076	0.0570
	R²	0.999999	0.999990	0.999997
Al-Cu-Zn_G77J6	MAE (%wt)	0.0612	0.0346	0.0169
	RMSE (%wt)	0.0947	0.0410	0.0262
	R²	0.999994	0.999999	0.999999

The results demonstrate that tree-based models are well-suited for rapid, accurate compositional analysis in LIBS applications. The ET model appears to be the most reliable regressor among the tested approaches when used with its default settings without the need for prior hyperparameter tuning. It exhibited the strongest bias control across different compositional regimes. The RF model offered a balanced compromise between accuracy and robustness. The GB model, however, proved to be less robust across broader compositional ranges.

The main limitation observed was that the models produced inaccurate predictions due to the insufficient representation of alloy families in the training dataset. Using only one sample per group does not provide enough variability for the models to capture the corresponding compositional domain, leading to inefficient learning and poor predictions. Our results indicate that reliable performance requires at least three representative samples from each group (e.g., cp-Al, Al–Cu, Al–Cu–Zn), enabling the models to interpolate more effectively across the expected compositional ranges. This finding highlights that ensemble regressors perform best in multi-element systems when supported by adequate diversity in the training data.

## Conclusion

The present work evaluated the accuracy of determining the elemental composition of cp-Al and its alloys by combining laser-induced breakdown spectroscopy (LIBS) with supervised machine learning models: RF, GB, and ET. The study concluded that tree-based regressors are adequate for LIBS applications, providing rapid, multi-element quantification with high accuracy across a broad range of compositions. The main conclusions are:

- The ET model showed the best overall performance, providing the most accurate predictions across all alloy types (MAE < 0.25 wt%, RMSE < 0.33 wt%).
- Model performance strongly depends on dataset diversity. Reliable predictions require at least three representative samples per alloy family to capture compositional variability.
- All models demonstrated good generalization capability, maintaining low prediction errors for independent samples; however, the ET model showed the most stable behavior when applied to unseen alloys.
- The GB model achieved more statistically indistinguishable results (*p* > 0.05); however, this was mainly due to higher prediction variance rather than improved predictive accuracy.

## Supplementary Information

Below is the link to the electronic supplementary material.

Supplementary Material 1

## Data Availability

The source code used in this work is openly available in Zenodo under the DOI: https://doi.org/10.5281/zenodo.15967233The archived version corresponds to the code used to generate all results presented in this study.
